# A brief induction of loving kindness meditation to reduce anti-fat bias

**DOI:** 10.1371/journal.pone.0302039

**Published:** 2024-06-20

**Authors:** Kristen M. Lee, Bita Ghanei, A. Janet Tomiyama

**Affiliations:** Department of Psychology, University of California Los Angeles, Los Angeles, California, United States of America; Teikyo University - Hachioji Campus: Teikyo Daigaku - Hachioji Campus, JAPAN

## Abstract

Weight stigma is highly prevalent. However, existing weight stigma interventions are only modestly effective at reducing anti-fat attitudes. The current research proposes a novel approach using a loving kindness meditation (LKM). Experiment 1 tests whether random assignment to the LKM intervention reduces explicit and implicit anti-fat bias and increases empathy based on the LKM recipient with higher weight (close other vs. stranger). Experiment 2 tests whether LKM outperforms an empathy intervention or control to increase empathy or reduce stigmatizing behavior. Results revealed that the LKM increased empathic care but did not reduce anti-fat bias compared to control; the LKM intervention, but not the empathy intervention, reported greater empathy compared to control in unadjusted analyses; and participants in the LKM and empathy interventions (vs. control) were more likely to engage in stigmatizing behavior. These findings suggest that the LKM may not be effective at reducing weight stigma despite increasing empathy.

## Introduction

Higher weight individuals are often socially devalued for their larger bodies and experience weight-based mistreatment known as weight stigma. Weight stigma targets individuals perceived to carry excess body weight and manifests as negative stereotyping, prejudice, or weight-based discrimination [1]. Previous research suggests that weight stigma may arise due to attributions of individual blame, such that higher weight individuals are viewed as predominantly responsible for their weight status, presumably due to a lack of willpower or self-control [2, 3]. Yet, there are a multitude of factors underlying higher weight status now recognized by the World Obesity Federation, who have recently issued a call to eliminate weight stigma [4].

Indeed, weight stigma is highly prevalent in the United States [5]. By one census-matched estimate, 42% of American adults have experienced weight stigma in their daily life [6]. Weight-based discrimination is highly pervasive in education, employment, and healthcare [3, 7]. Individuals with obesity are frequently viewed as “lazy,” “stupid,” and “worthless,” even by healthcare professionals who specialize in obesity treatment [8]. Extensive research shows that weight stigma predicts poorer mental health outcomes, including body dissatisfaction, depression, anxiety, and psychological distress [9].

Moreover, weight stigma is physically harmful. There is strong evidence that weight stigma functions as a stressor that increases physiological and psychological stress [1]. Other work has shown that weight stigma is linked to multiple poorer health behaviors, including alcohol use and poor sleep [6], and all-cause mortality, controlling for BMI [10]. Controlling for BMI is important as it is a potential confound that could cause both weight stigma experiences and poorer outcomes [7, 11]. Despite the considerable harm of weight stigma, reliably reducing anti-fat attitudes has proven difficult with existing intervention approaches [12–14]. As such, the present research proposes an alternative weight stigma intervention based on loving kindness meditation (LKM).

Current weight stigma interventions tend to adopt one of three approaches: shifting beliefs about the controllability of weight, targeting social norms, or generating empathy. Controllability interventions target cognitive beliefs about higher weight status through emphasizing the lack of control over weight or by highlighting the myriad factors (e.g., genetics) that determine one’s body weight [15]. In comparison, social consensus interventions attempt to signal social norms that endorsing positive beliefs about higher weight status is more socially acceptable than denigrating obesity [16]. Alternatively, as the name suggests, empathy interventions aim to reduce weight stigma by evoking empathy for higher weight individuals through narratives of experienced stigma and hardship [17, 18]. Yet, research suggests that such approaches are modestly effective [14], yielding only small to medium effects on reducing weight-biased attitudes or beliefs [13]. Indeed, some suggest that empathy interventions may inadvertently reinforce negative aspects of being higher weight by depicting individuals as pitiable, thereby confirming negative stereotypes of being weak-willed or lacking in self-control. Thus far, it remains unclear if increasing feelings of empathy alone may be counterproductive at reducing weight stigma. In the same vein, calls to reduce weight bias emphasize the need for new strategies and more experimental work [12].

In recent years, the contemplative practice of LKM [19] has been tested as an alternative approach to reducing bias towards other stigmatized groups. Previous work has found that LKM, which some consider to be a type of mindfulness meditation, reduces intergroup bias [20]. Unlike more commonly known mindfulness meditations that ask individuals to adopt a neutral stance towards bodily or external experiences, the LKM is intended to generate positive feelings of love and concern for self and others. LKM is performed by bringing to mind a subject and repeating a series of statements (e.g., “May you be at ease”; “May you be at peace”) whether aloud or silently. That is, the goal of LKM is to foster a positive affective state in which you wish warm regard for others thereby increasing empathy [21]. Indeed, research shows that the LKM significantly increases positive emotions and relations with others [22, 23]. Furthermore, while traditional empathy approaches tend to induce pity through narratives of discrimination and mistreatment [17, 18, 24], the LKM places greater emphasis on generating feelings of acceptance, equality, and respect for others, which some have suggested may have greater promise [14].

For example, one randomized controlled trial found that participants who performed a brief LKM directed towards a racial out-group reduced their implicit racial bias compared to those in the control group [25]. Research also shows that LKM can increase feelings of social connectedness and positivity towards strangers [26] and simply exposing participants to loving kindness statements (e.g., “May you be at ease”) can increase their sensitivity towards others’ experiences [27]. To our knowledge, no studies have tested LKM to reduce explicit or implicit anti-fat attitudes towards stigmatized higher weight individuals.

To fill this gap, Experiment 1 first tested LKM as a brief weight stigma intervention to reduce explicit and implicit anti-fat bias towards higher weight individuals and increase empathy using vignettes. We also examined whether the effects of a LKM intervention would differ based on the strength of participants’ social ties to the recipient (i.e., close others vs. strangers). Investigating the strength of social ties is critical as research suggests that individuals do not report lower anti-fat bias when they themselves have experienced weight stigma but will when their family or friends have been stigmatized for their weight [28]. We wondered whether this effect required having a personal relationship to the stigmatized other, and therefore tested strangers.

We predicted that participants in the LKM condition would report lower explicit and implicit anti-fat bias compared to those in the control condition. We also predicted that participants in the LKM condition would report greater feelings of empathy compared to those in the control condition, as measured by feelings of tenderness, perspective taking, and lower distress. We predicted that the effects of the LKM on anti-fat bias would be larger for close others than strangers. Experiment 2 tested whether participants in the LKM would report lower explicit anti-fat bias and greater empathy compared to a general empathy intervention or control. We also tested whether those in the LKM would engage in less stigmatizing behavior towards higher weight individuals compared to those in the other two conditions.

## Materials and methods

All hypotheses, measures, and analytic plans were pre-registered and are available on the OSF page (Study 1: https://osf.io/639qc?view_only=b03baf3b77414ebcaa0b25e9b69aedb7; Study 2: https://osf.io/qrkma?view_only=95dcf66295044301bf435ad4f3c625c0). The current studies received IRB approval (IRB#21–00212) by the North General Institutional Review Board (NGIRB) and were conducted at the same university in the United States. The authors did not have access to information that could identify individual participants after data collection.

### Experiment 1

#### Design

The present study was a 2 (condition: LKM vs. control) x 2 (recipient: close other vs. stranger) between-subjects randomized experiment conducted online using *Qualtrics* software.

#### Participants

Eligible participants were recruited from a large university in the U.S through the subject pool between January to February 2022. Participants (*N* = 395) were compensated for their time (*M* = 24.43 minutes) with 0.5 research credits. The target sample size was determined based on the maximum possible number of study hours from the subject pool and data collection was terminated at the end of the academic quarter. Sample size was determined before any data analysis. Individuals were required to be at least 18 years of age and English speaking/reading. Participants who did not follow study instructions (*n* = 44) or failed the manipulation check (*n* = 7) were excluded from the analyses. There were no significant differences in baseline characteristics found with the excluded sample apart from a slightly higher education (*M* = 3.69) than the valid sample (*M* = 3.29), *p* = .042. Randomization was successfully executed as there were no significant differences across demographic variables between conditions (*p*s > .05). Experiment 1 had 344 participants in the analytic sample. Descriptive sample statistics are provided in Table 1.

**Table 1:** Sample characteristics for Experiment 1 (*N* = 344).

Characteristic		*n*	(%)
Gender			
	Woman	283	82.3
	Man	55	16
	Prefer to self-describe	2	0.6
	Prefer not to stay	4	1.2
Race/Ethnicity			
	Asian or Pacific Islander	144	42
	White	107	31.2
	Hispanic, Latino	54	15.7
	Biracial/Multiracial	17	5.0
	Other	12	3.5
	Black, African American	7	2
	Native American, Eskimo, Aleut	2	0.6
Education			
	Some high school/GED	109	31.8
	Two-year college, Associates degree	82	23.8
	Four-year college, Bachelor’s degree	136	39.7
	Masters degree	0	0
	Professional degree (e.g., PhD, MD]	1	0.3
	Prefer not to answer/don’t know	15	4.3
Sexual orientation			
	Heterosexual	272	79.1
	Homosexual	16	4.7
	Bisexual	37	10.8
	Prefer not to say	15	4.4
	Another sexual orientation	4	1.2

*Note*. Education missing 1, race/ethnicity missing 1. Education refers to highest level of the participant’s education.

#### Measures

*Demographics*. Participants were asked to report demographic information including age, gender, marital status, sexual orientation, race/ethnicity, education, and parental income.

*Explicit anti-fat bias [29].* We assessed explicit anti-fat bias using the Fat Phobia Scale–Short Form, which assesses attitudes about higher weight individuals using 14 pairs of antonyms (e.g., “lazy” vs. “industrious”), anchored at each end point of a 5-point Likert scale. Participants were instructed to indicate the adjective that best describes a person with obesity. Scores were summed to compute a total explicit anti-fat bias, with higher scores indicating greater anti-fat bias. Cronbach’s alpha = 0.88.

*Implicit association test [30]*. To measure implicit anti-fat attitudes, we used the IAT, which measures reaction times as participants classify “fat” vs. “thin” people with positive vs. negative attributes. An IAT D-score is calculated by taking the relative mean difference in response times between the congruent and incongruent trial blocks, with a negative D-score reflecting a larger implicit anti-fat, pro-thin bias.

*Empathy [31, 32].* We assessed empathy using a 7-point Likert scale (1 –“Not at all” to 7 –“Extremely/Very much so”), in which participants responded to three items that captured different components of empathy: empathic care (e.g., how tender do you feel right now? By ‘tender’ we mean a feeling of caring, openness, and warmth toward another person), empathic distress (e.g., how distressed do you feel right now? By ‘distressed,’ we mean a feeling of discomfort or suffering for you), and perspective taking (e.g., did you imagine how you would feel in the situation described in the vignette?) based on validated scales.

#### Procedures

After reading an information sheet, participants were randomly assigned to either a “Close Other” condition or “Stranger” condition. In both conditions, participants read identical vignettes describing a weight-stigmatizing scenario (see Appendix A in S1 File) in which an individual is refused to be shown clothes by a salesperson because of their larger body size. However, participants who were randomly assigned to the “Close Other” condition were asked to visualize a friend with higher weight and imagine their friend as the individual in this hypothetical scenario. Participants randomly assigned to the “Stranger” condition were asked to visualize the higher weight person “Alex” described in the hypothetical situation.

Then, participants were randomly assigned to either a three-minute LKM intervention or a control condition. The 3-minute dosage and design of the LKM intervention were chosen to reduce participant burden based on previous studies using brief meditation interventions [25, 33]. In the LKM condition, participants were instructed to write a message to their recipient (close other or stranger) showing concern about the stigmatizing scenario and wishing them health, happiness, and wellbeing. Notably, they were specifically asked to include four LKM phrases in their message, which were provided to them in the instructions (“may you be safe,” “may you be happy,” “may you be healthy,” “may you live with ease”). Participants randomly assigned to the control condition were given a writing prompt that asked them to recall the scenario and describe what happened in their own words step by step. They were given four phrases (i.e., “mall,” “clothing store,” “higher weight,” and “pair of pants”) to include in their message to parallel the writing instructions in the LKM condition. The online writing task was programmed to automatically advance to the next section of the study after 3-minutes.

Afterwards, all participants completed questionnaires on empathy and explicit anti-fat attitudes as well as an implicit associations test. At the end of the study, participants were debriefed by a research assistant.

## Results

The final analytic sample comprised 344 participants (*Mage* = 20.71, *SD* = 3.15). See Table 1 for sample descriptive statistics. Table 2 presents a bivariate correlation matrix of all study variables in Experiment 1.

**Table 2:** Correlation matrix of study variables in Experiment 1 (*N* = 344).

	1	2	3	4	5
1. Explicit anti-fat bias	-				
2. Implicit anti-fat bias	-.17**	-			
3. Empathic care	-.20**	.03	-		
4. Perspective-taking	-.06	.03	.27**	-	
5. Empathic distress	-.09	-.02	.22**	.28**	-

Note.

* p < .05

** p < .01

### Explicit anti-fat bias

A two-way analysis of variance (ANOVA) revealed no statistically significant interaction effect between the recipient (close other vs. stranger) and condition (LKM vs. control) on explicit anti-fat bias, *F*(1, 340) = 3.66, *p* = .057, *η*_*p*_^*2*^ = 0.01. Moderation analyses using PROCESS macro (model #1; Hayes, 2022) further confirmed that there was no significant moderation effect of the type of recipient, *b*_3_ = 0.23, 95% CI [-0.01, 0.47], *t*(340) = 1.91, *p* = .057. There was no significant main effect of condition, *F*(1, 340) = 3.34, *p* = .068, *η*_*p*_^*2*^ = 0.01. There was, however, an unhypothesized significant main effect of recipient such that participants who brought to mind a close other with higher weight reported less explicit anti-fat bias compared to those who brought to mind a stranger with higher weight, *F*(1, 340) = 3.89, *p* = .049, *η*_*p*_^*2*^ = 0.01. See Table 3 for descriptive statistics per experimental condition.

**Table 3:** Means and standard deviations for anti-fat bias outcomes by condition x recipient.

	*Control x* *Stranger*			*LKM x* *Stranger*			*Control x* *Close Other*				*LKM x* *Close Other*		
	*n*	*M*	*SD*	*n*	*M*	*SD*	*n*	*M*		*SD*	*n*	*M*	*SD*
Implicit Bias	95	-0.51	0.38	84	-0.43	0.46	83	-0.53	0.40		82	-0.49	0.42
Explicit Bias	95	3.47	0.48	84	3.24	0.58	83	3.23	0.59		82	3.24	0.58

### Implicit anti-fat bias

A two-way ANOVA revealed no statistically significant interaction between condition and type of recipient on IAT *D*-scores, *F*(1, 340) = 0.31, *p* = .577, *η*_*p*_^*2*^ = 0.001. Moderation analyses further confirmed that there was no significant moderation effect by type of recipient, *b*_3_ = -0.05, 95% CI [-0.23, 0.13], *t*(340) = -0.56, *p* = .577. There was no significant main effect by condition, *F*(1, 340) = 1.85, *p* = .174, *η*_*p*_^*2*^ = 0.01, indicating that the LKM intervention did not reduce implicit anti-fat bias among participants compared to control. There was also no significant main effect of type of recipient, *F*(1, 340) = 0.84, *p* = .359, *η*_*p*_^*2*^ = 0.002, such that there was no difference in implicit anti-fat bias between participants who brought to mind a close other and those who brought to mind a stranger. A one sample *t*-test revealed that participants across conditions showed significant implicit anti-fat bias with a mean IAT score of -0.48, which was significantly different from zero, *t*(275) = -19.26, *p* < .001, 95% CI_*D*-score_ [-0.53, -0.43], *d* = -1.16. See Table 3 for descriptive statistics per experimental condition.

### Empathic care *(i*.*e*., *feelings of caring*, *openness*, *and warmth towards others)*

The results of a two-way ANOVA revealed only a statistically significant main effect of condition, indicating that participants in the LKM intervention reported significantly greater empathic care compared to those in the control condition, *F*(1, 340) = 15.96, *p* < .001, *η*_*p*_^*2*^ = 0.05. This main effect of condition was not qualified by an interaction with type of recipient, *F*(1, 340) = 2.38, *p* = .124, *η*_*p*_^*2*^ = 0.01. There was also no significant main effect of recipient on empathic care, *F*(1, 340) = 0.25, *p* = .621, *η*_*p*_^*2*^ = 0.001.

### Empathic distress *(i*.*e*., *feelings of discomfort or suffering for oneself)*

The results of a two-way ANOVA revealed no statistically significant interaction effect, *F*(1, 340) = 0.02, *p* = .880, *η*_*p*_^*2*^ < .001, no main effect of type of recipient, *F*(1, 340) = 2.98, *p* = .085, *η*_*p*_^*2*^ = 0.01, and no main effect of condition on empathic distress, *F*(1, 340) = 2.34, *p* = .127, *η*_*p*_^*2*^ = .01.

### Perspective-taking *(i*.*e*., *imagining oneself in the situation)*

The results of a two-way ANOVA revealed no statistically significant interaction effect, *F*(1, 340) = 3.26, *p* = .072, *η*_*p*_^*2*^ = 0.01, no main effect of type of recipient, *F*(1, 340) = 1.29, *p* = .256, *η*_*p*_^*2*^ = 0.004, and no main effect of condition on perspective taking, *F*(1, 340) = 0.43, *p* = .513, *η*_*p*_^*2*^ = .001.

Please refer to Table 4 for descriptive statistics on all empathy measures.

**Table 4:** Means and standard deviations for empathy by condition.

		LKM intervention		Control		
	*n*	*M*	*SD*	*n*	*M*	*SD*
Empathic Care	166	5.20	1.23	178	4.63	1.43
Empathic Distress	166	3.70	1.62	178	3.96	1.65
Perspective-taking	166	5.46	1.72	178	5.37	1.60

### Experiment 2

To determine whether the LKM intervention was uniquely effective compared to a typical empathy intervention, Experiment 2 introduced a third condition, in which participants were asked to provide general support to their stigmatized close other after reading the vignette. All participants were asked to bring to mind a close other with higher weight. To improve clarity about the target of empathy, Experiment 2 specified higher weight individuals and tested whether the LKM led to less stigmatizing behavior. These pre-registered mediation analyses appear in the supporting information (Appendix D in S1 File). We hypothesized that participants in the LKM condition would report higher feelings of empathy towards higher weight individuals and lower body shame compared to those in the empathy or control conditions. In terms of less stigmatizing behavior, we expected that participants in the LKM condition would be more likely to reward a wellbeing membership gift card rather than a weight loss membership (the operationalization of stigmatizing behavior) to higher weight individuals than those in the empathy or control conditions. We expected that individuals who engaged in stigmatizing behavior would report higher levels of explicit anti-fat bias than those who engaged in less stigmatizing behavior.

#### Participants

Eligible participants were recruited at the same university in the U.S through the subject pool between May to June 2022. Individuals were required to be at least 18 years of age and English speaking/reading. Any individual who had completed Experiment 1 was deemed ineligible to participate. Participants (*N* = 198) were compensated for their time (*M* = 25.68 minutes) with 0.5 research credits. Target sample size was determined using the same criteria as Experiment 1 prior to data analysis. Participants who did not follow study instructions (*n* = 30) were excluded, leading to 168 participants as the final analytic sample. There were no significant differences in baseline characteristics found with the excluded sample. Randomization was successful with no differences between the LKM, empathy, or control conditions across variables (age, race, education, gender, sexual orientation), *p*s > .05.

#### Measures

Experiment 2 used the same explicit anti-fat bias measure as Experiment 1. We refined the empathy measure in Experiment 2 to focus on empathic care based on Experiment 1’s outcomes using a measure that incorporated distractor items. We also improved clarity in the questionnaire by explicitly stating that the feelings were directed towards higher weight individuals, as described below.

*Explicit anti-fat bias [29].* We assessed explicit anti-fat bias using the Fat Phobia Scale–Short Form, which assesses attitudes about higher weight individuals using 14 pairs of antonyms (e.g., “lazy” vs. “industrious”), anchored at each end point of a 5-point Likert scale. Participants were instructed to indicate the adjective that best describes a person with obesity. Scores were summed to compute a total explicit anti-fat bias, with higher scores indicating greater anti-fat bias. Cronbach’s alpha = 0.89.

*Empathy [18, 32].* To assess empathy, participants were asked to rate 14 adjectives to describe their current emotional state toward higher weight individuals, which included six empathy items (i.e., sympathetic, soft-hearted, warm, compassionate, tender, and moved) and eight distractor items. A mean empathy score of the six items was computed. Based on the findings from Experiment 1 regarding empathic care, we chose a measure designed to more accurately gauge feelings of tenderness. To ensure the participants remained unaware of our focus on empathy, we incorporated distractor items into the measure. We also changed the question wording to ensure that participants were rating their feelings towards higher weight individuals specifically. Cronbach’s alpha = 0.83.

*Body shame [34].* To assess body shame, the Body Shame subscale from the Objectified Body Consciousness Scale was used (e.g., “I would be ashamed for people to know what I really weigh”). Items were rated on a 7-point Likert scale (1 –Strongly disagree to 7 –Strongly agree) and a mean body shame score was computed. Cronbach’s alpha = 0.82.

*Stigmatizing behavior*. To include a behavioral outcome, stigmatizing behavior was assessed as the selection of a gift card option for higher weight individuals that was either for a weight loss membership program (stigmatizing behavior) or a wellbeing spa membership program (less stigmatizing behavior). *Weight Watchers* and *Noom Weight Loss* gift card choices were coded as 0 to indicate stigmatizing behavior, whereas the *Burke Williams* or *NOW* Spa massage gift cards were coded as 1 to indicate less stigmatizing behavior. Although the general public may view gifting a higher weight individual with a weight loss program as helpful, weight-loss related messaging is often regarded as stigmatizing by higher weight individuals [35, 36]

#### Procedure

The procedures in Experiment 2 were identical to Experiment 1 with minor modifications. First, all participants were asked to bring to mind a friend of higher weight before reading a vignette describing a weight-stigmatizing scenario in which an individual is refused to be shown clothes because of their larger body size. If the participant did not have a higher weight friend, they were asked to bring to mind a close acquaintance with higher weight. A manipulation check was included to ensure that participants brought to mind a close other with higher weight. Participants were asked to report what type of person they brought to mind by selecting from the following options (friend with higher weight, friend with average weight, close acquaintance with higher weight, family member with higher weight).

Then, participants were randomly assigned to either a 3-minute LKM intervention, empathy intervention, or a control condition. The LKM and control conditions were identical to Experiment 1. In the empathy intervention, participants were asked to write a message about the situation and give support. They were given the same four phrases (i.e., “mall,” “clothing store,” “higher weight,” and “pair of pants”) to include in their message as the control condition to parallel the writing instructions of the LKM intervention. The empathy intervention diverges from the LKM intervention in its approach, as it refrains from directing participants to focus on specific content or adopt particular affective orientations. Instead, it asks participants to offer support, in whatever manner they desire (e.g., reassurance, disapproval of the manager, suggestions to shop elsewhere, pity). In contrast, the LKM method is characterized by its precision, as it instructs participants to extend goodwill towards others from a foundation of respect through prescribed phrases. See Appendix B in S1 File for the written instructions per experimental condition and sample responses. After completing the writing task, participants completed measures on empathy, explicit anti-fat attitudes, and body shame.

At the end of the study, prior to debriefing, participants were led to believe that another graduate student in the lab was fielding opinions on the type of gift card (weight loss vs. wellbeing) they should offer to a higher weight sample as part of their dissertation project. See Appendix C in S1 File for experimental stimuli. The order of gift card appearance was randomly displayed. At the end of the study, participants were debriefed.

## Results

The final analytic sample comprised 168 participants (*Mage* = 20.20, *SD* = 2.57). See Table 5 for descriptive statistics. Table 6 presents a bivariate correlation matrix of all study variables in Experiment 2.

**Table 5:** Sample characteristics for Experiment 2 (*N* = 168).

Characteristic		*n*	(%)
Gender			
	Woman	130	77.4
	Man	36	21.4
	Nonbinary	2	1.2
Race/Ethnicity^1^			
	Asian or Pacific Islander	62	36.9
	White	48	28.6
	Hispanic, Latino	30	17.9
	Biracial/Multiracial	15	8.9
	Black, African American	8	4.8
	Native American, Eskimo, Aleut	3	1.8
	Other	1	1.2
Education			
	Some high school/GED	59	35.1
	Two-year college, Associates degree	24	14.3
	Four-year college, Bachelor’s degree	77	45.8
	Masters degree	1	0.6
	Prefer not to answer	7	4.2
Sexual orientation			
	Heterosexual	129	76.8
	Homosexual	7	4.2
	Bisexual	27	16.1
	Prefer not to say	5	3.0

*Note*. Education missing 1, race/ethnicity missing 1. Education refers to highest level of the participant’s education.

**Table 6:** Correlation matrix of study variables in Experiment 2 (*N* = 168).

	1	2	3	4
1. Anti-fat bias	-			
2. Empathy	-.17*	-		
3. Body Shame	.03	.00	-	
4. Less stigmatizing behavior	.10	-.21**	.04	-

Note.

* p < .05

** p < .01

### Empathy towards higher weight individuals

A one-way ANOVA revealed no statistically significant differences in reported empathy across conditions, *F*(2, 165) = 2.49, *p* = .086, *η*^*2*^ = 0.03. Pairwise comparisons revealed that participants in the LKM intervention did not report significantly greater empathy compared to the empathy condition, *p* = .659. However, those in the LKM intervention reported significantly greater empathy compared to those in control, *p* = .037.

### Body shame

A one-way ANOVA revealed no statistically significant differences in reported body shame across conditions, *F*(2, 165) = 0.04, *p* = .962, *η*^*2*^ < 0.001. Pairwise comparisons revealed that participants in the LKM condition did not report significantly less body shame compared to the empathy condition, *p* = .969. Similarly, the LKM condition did not report significantly less body shame compared to those in control, *p* = .828. No differences in body shame also emerged between empathy and control conditions, *p* = .794.

See Table 7 for descriptive statistics and Table 8 for adjusted and unadjusted analyses.

**Table 7:** Means and standard deviations for Experiment 2 outcome variables.

	LKM condition			Empathy condition			Control condition		
	*n*	*M*	*SD*	*n*	*M*	*SD*	*n*	*M*	*SD*
Empathy	55	4.41	1.28	59	4.31	1.31	54	3.91	1.11
Body Shame	55	3.94	1.11	59	3.93	1.23	54	3.99	1.24

**Table 8:** Planned pairwise comparisons for Experiment 2 outcome variables.

	Comparison						
Outcome	Condition	Condition	Mean Difference	SE	95% *CI*	*p*	*pTukey*
Empathy	LKM	Support	0.10	0.23	-0.36, 0.56	.659	.898
	LKM	Control	0.50	0.24	0.03, 0.97	.037	.091
	Control	Support	-0.40	0.23	-0.86, 0.06	.090	.206
Body Shame	LKM	Support	0.01	0.22	-0.43, 0.45	.969	.999
	LKM	Control	-0.05	0.23	-0.50, 0.40	.828	.974
	Control	Support	0.06	0.23	-0.39, 0.50	.794	.963

### Stigmatizing behavior

In general, significantly more participants selected wellbeing spa gift cards (*n* = 127) for higher weight individuals rather than weight loss gift cards (*n* = 33). A binary logistic regression analysis was used to test the effect of the LKM intervention on the likelihood of engaging in stigmatizing behavior. For this set of analyses, the control condition was used as the reference group, the LKM intervention was coded as 1 and the empathy condition was coded as 2. To facilitate interpretations, the stigmatizing behavior of gifting a weight-loss gift card was coded as 1. The binary logistic regression was statistically significant, χ2(2, *N* = 160) = 9.04, *p* = .011. The odds of participants in the LKM intervention choosing a weight loss gift card for higher weight individuals were 4.62 times greater than those in control, *B* = 1.53, *SE* = 0.60, *OR* = 4.62, 95% CI (1.42, 15.04), *p* = .011. Similarly, the odds of participants in the empathy condition gifting a weight loss membership gift card to higher weight individuals were 4.20 times greater than those in control, *B* = 1.44, *SE* = 0.61, *OR* = 4.20, 95% CI [1.28, 13.78], *p* = .018.

### Explicit anti-fat bias

An independent samples *t*-test revealed that participants who engaged in stigmatizing behavior did not report significantly greater anti-fat bias (*M* = 3.36, *SD* = 0.65) compared to those who engaged in less stigmatizing behavior (*M* = 3.27, *SD* = 0.60), *t*(158) = -0.69, *p* = .491, *d* = -0.14. Despite the large group size difference between the less stigmatizing behavior group (*n =* 127) and the stigmatizing behavior group (*n* = 33), the Levene’s Test for Equality of Variances indicated that homogeneity of variance had not been violated, *F* = 0.336, *p* = .563.

## Discussion

The current studies sought to test an alternative weight stigma intervention based on the contemplative practice, LKM. We found that participants in the LKM intervention were more likely to report greater tender feelings of empathy toward higher weight individuals compared to those in the control condition (Experiments 1 and 2) but not the empathy intervention (Experiment 2), regardless of whether the higher weight recipient was a close other vs. stranger (Experiment 1). Contrary to our hypotheses, participants in the LKM intervention did not report significantly lower explicit (Experiments 1 and 2) or implicit anti-fat bias (Experiment 1) compared to control, nor did they report significantly lower body shame compared to the empathy intervention or control (Experiment 2). Furthermore, participants in the LKM and empathy conditions had *greater* odds of engaging in weight stigmatizing behavior compared to those in the control condition (Experiment 2). That is, participants were more likely to choose a weight loss membership for higher weight individuals instead of a wellbeing membership.

Although burgeoning work on LKM has shown successful reductions in implicit bias across other marginalized groups [25, 37], we did not find evidence of this in the present investigation on weight stigma. One possible explanation for this may be the LKM paradigm used in the current study, which asked participants to perform a written LKM for a higher weight recipient. Conversely, the brief LKM intervention in Stell and Farsides’ (2016) randomized controlled trial (*N* = 69) had been performed in-person. That is, participants in the LKM intervention were asked to bring to mind individuals who cared deeply for them for four minutes before directing the elicited feelings and LKM phrases to an image of a gender-matched Black stranger. Similarly, Kang and colleagues (2014) observed no between-group differences in explicit bias towards Black individuals or unhoused people, but did find a significant reduction in implicit bias towards Black individuals and unhoused people in the LKM practice intervention compared to those in the waitlist control or LKM discussion conditions. Albeit their LKM practice intervention took place over the course of six weeks and included a weekly sitting meditation class led by an accredited instructor, group-based discussion, and a regular at-home LKM practice.

Additionally, it may be that the LKM intervention was not as effective at reducing weight bias compared to racial bias from previous studies because obesity is often viewed as a personal failure [38, 39], which is reinforced by existing obesity-related policies that promote stigmatizing discourse [40]. Taken together, the contemplative practice of LKM shows some promise at shifting enduring implicit biases towards stigmatized groups, however future research should test whether more intensive forms of a LKM intervention can successfully reduce enduring anti-fat attitudes towards higher weight individuals.

One unexpected finding in the current study was that participants in the LKM intervention had reported greater feelings of empathy towards higher weight individuals compared to control, yet they were also more likely to engage in weight-stigmatizing behavior than those in the control condition. One possibility for the present finding is that participants believed that encouraging weight loss was an act of kindness by gifting “good health.” Indeed, weight loss has been promoted both by physicians [41] and popular media [42] as ways to improve physical health and self-esteem, despite limited evidence that weight loss is sustainable in the long-term [43] or necessary for health [44]. Research also suggests that some higher weight individuals may find the dominant weight loss discourse stigmatizing [36]. Our preliminary data suggest that even with the best intentions, individuals may still stigmatize their higher weight family or friends in their misguided attempts to help.

Alternatively, these findings might imply that heightened empathy does not alter the underlying stereotypes regarding higher weight or weight loss, and thus does not mitigate stigmatizing behavior. Indeed, Experiment 1 found that all participants, regardless of condition, demonstrated strong implicit anti-fat bias. Similarly, Teachman and colleagues (2003) conducted an experiment in which participants were asked to read about a stigmatizing scenario encountered by a higher weight individual. Even though the empathy condition effectively elicited empathy among participants, there was no significant difference observed in implicit or explicit bias when compared to those in the control condition. However, the study revealed that among participants with *higher weight*, those in the empathy condition exhibited lower levels of anti-fat bias compared to those in the control condition. Potentially, the LKM may be more effective in mitigating internalized weight stigma among individuals with higher weight, though more research is needed.

Moreover, given that the recipient of the gift card was not the same individual brought to mind during the LKM intervention, but rather an unfamiliar group of higher weight individuals, it is possible that the feelings of empathy did not transfer over. In the same vein, previous work has shown that hypothetical stories that evoke feelings of empathy are not significantly related to prosocial behavior compared to other indices of empathy [45]. Future research is needed to determine whether weight stigmatization still occurs when the target of empathy and supportive behavior remain the same, and whether other indices of empathy (e.g., empathy-evoking experimental stimuli based on reality and not hypotheticals) may lead to less stigmatizing behavior towards higher weight individuals.

As a final point, we found that participants who brought to mind a close other with higher weight reported lower explicit anti-fat attitudes compared to those who thought of a higher weight stranger. This finding corroborates previous work showing that individuals are less likely to report anti-fat attitudes when their family or friends have experienced weight stigma [28]. Given that this difference did not emerge with the implicit anti-fat attitudes measure, it is possible that individuals were simply less willing to disclose their explicit bias despite endorsing those beliefs internally. Indeed, other research suggests that family members tend to be some of the most stigmatizing [46]. The conflicting evidence suggests that it remains important to examine the strength of social ties in the context of weight stigma.

### Limitations and future directions

There are limitations to consider in the present study. First, the LKM intervention was tested as a weight bias intervention among two university student samples of predominantly young women who identified as white or Asian. Some research suggests that women are more likely to internalize weight stigma [5] and therefore may be more sensitive to the stigma of weight loss messaging, thus inflating the observed effects. However, the selection of weight loss gift cards may have been even higher among more diverse community samples, who have been shown to be more likely to recommend weight-based behaviors (e.g., diet, count calories) compared to student samples [47]. Future research should test the effectiveness of the LKM intervention in community samples, testing potentially important perceiver moderators such as weight and gender.

Additionally, the brief induction of the LKM intervention was conducted online via a written exercise. Although the intervention is based on the LKM and incorporates key elements of the practice, the exercise itself lacks an active meditative practice found in other studies. Future research should test different “doses” of the current LKM paradigm with more active meditative components to determine the role of LKM in weight stigma interventions. A stronger dosage than 3-minutes may be warranted to observe changes in explicit and implicit bias.

Lastly, we tested LKM as an alternative weight stigma intervention for its potential to circumvent the challenges of traditional empathy interventions, which have been shown to be largely ineffective at reducing weight stigma [14]. Although the LKM intervention was tested in the present investigation, there may be other forms of meditation such as mindfulness meditation that may be more effective at reducing weight stigma. Considering the novelty of our LKM intervention, we caution against using the current findings as evidence that empathy-evoking interventions are harmful. Rather, we suggest that future research test other contemplative practices or extended models of the LKM.

## Conclusion

To our knowledge, the current experiments are the first to test a LKM intervention to reduce anti-fat bias towards higher weight individuals. The present findings suggest that a brief LKM intervention may be effective at increasing empathic feelings towards higher weight individuals, but not at reducing explicit or implicit anti-fat bias or weight-stigmatizing behaviors. More generally, our findings provide further supporting evidence that empathy-based interventions may be less effective at combating weight stigma [14].

## Supporting information

S1 File Appendices A–D provide additional information on the study instructions, experimental stimuli, and mediation analyses. (DOCX)
