# Supplementary material for: A brief induction of loving kindness meditation to reduce anti-fat bias
Source: PLoS One. 2024 Jun 20;19(6):e0302039. doi: 10.1371/journal.pone.0302039 (PMC11189228; doi:10.1371/journal.pone.0302039)
Supplement: S1 File — (DOCX) [file pone.0302039.s001.docx]

**Appendix A**

Study Instructions

***Close Other^[[1]](#footnote-1)^* vignette (Experiment 1 and 2):**

Please take a moment to bring to mind a **friend with higher weight** **(i.e., considered having an overweight or obese BMI).** If you cannot think of a friend with higher weight, please think of a close acquaintance with higher weight.

As you read the vignette on the next page, please imagine this person in the scenario.

Last weekend, your friend with higher weight went shopping for new pants at the mall. They entered one of their favorite clothing stores and began browsing the clothing racks.

Shortly after, one of the store workers approached them and asked if they were looking for anything in particular. When your friend shared that they were looking for a new pair of pants, the store worker responded with: “Unfortunately, I’m afraid we don’t carry anything in your size in our store. It would also be difficult for you to return the clothing if you decided to return anything, since we can only accept clothing in their original condition. I think there is a plus-size collection at the store next door that you could try instead.”

Your friend left the store feeling mortified and ended their shopping early.

**Stranger vignette (Experiment 1):**

As you read the vignette on the next page, please imagine the **person with higher weight (i.e., considered having an overweight or obese BMI)**in the scenario.

Last weekend, an individual with higher weight named Alex went shopping for new pants at the mall. They entered one of their favorite clothing stores and began browsing the clothing racks.

Shortly after, one of the store workers approached them and asked if they were looking for anything in particular. When Alex shared that they were looking for a new pair of pants, the store worker responded with: “Unfortunately, I’m afraid we don’t carry anything in your size in our store. It would also be difficult for you to return the clothing if you decided to return anything, since we can only accept clothing in their original condition. I think there is a plus-size collection at the store next door that you could try instead.”

Alex left the store feeling mortified and ended their shopping early.

**Appendix B**

Study Instructions

**LKM intervention (Experiments 1 and 2)**

Now, please bring to mind the person from the vignette you just read and write a message to them about the situation and wish them health, happiness, and wellbeing in the text box below.

Please include all of the following phrases in your message:

May you be safe.

May you be happy.

May you be healthy.

May you live with ease.

You will have three minutes to complete this task. **Please write continuously for the full duration of the task.** Please do not worry about grammar, spelling, or formatting. The page will automatically advance after 3 minutes.

**Empathy message (Experiment 2)**

Now, please bring to mind the person from the vignette you just read and write a message to them about the situation and give them support in the text box below.

Please include all of the following phrases in your message:

Mall

Clothing store

Higher weight

Pair of pants

You will have three minutes to complete this task. **Please write continuously for the full duration of the task.** Please do not worry about grammar, spelling, or formatting. The page will automatically advance after 3 minutes.

**Control (Experiments 1 and 2)**

Now, please bring to mind the person in the vignette you just read and recount what happened in the situation step by step in your own words in the text box below. Please write as objectively as possible.

Please include all of the following phrases in your message:

Mall

Clothing store

Higher weight

Pair of pants

You will have three minutes to complete this task. **Please write continuously for the full duration of the task.** Please do not worry about grammar, spelling, or formatting. The page will automatically advance after 3 minutes.

**Real example responses collected from the general Empathy condition:**

1. *“You shouldn't have to look for another mall neither another clothing store. This clothing store should have a section for people with higher weights. Not only light weight people wears a pair of pants.”*
2. *“Hey! I heard about what happened to you at the clothing store at the mall. I'm so sorry that people were so rude to you. You should be able to go into any store without being treated rudely like that. I hate that they made it seem like buying a pair of pants was such a big deal. Please don't let them make you fall bad about it. I can't believe they implied that you would stretch them out like that because you have a higher weight. Its so disrespectful to talk to someone like that. I hope you know that you are amazing and beautiful and no one should talk down to you for any reason.”*

**Real example responses collected from the LKM condition:**

1. *LKM: “May you be safe in your future endeavors. May you be happy. May you be healthy. May you live with ease. I hope you go on to live without a worry and find yourself comfortable with yourself and your own skin. May you find joy in things that bring you the most happiness. Do not let others get in the way of you finding your passion for life and seeking out the flavors of life. Enjoy the small things and cherish them like the last drop of water on earth. You are a human being like anyone else and you deserve to be here like everyone else. Beauty is in the eye of the beholder and you are beautiful in every single way.”*
2. *“Even though you do not feel secure in yourself. May you be safe. May you be happy. Do it in spite of others. The most important factor is your own health. May you be healthy. May you live with ease. You do not deserve to be faced with barriers that are unfair and biased. Live within yourself and be the best version of yourself that you could choose to be, despite the opinions of others and their incorrect judgments. Clothes are made to fit you, you are not made to fit the clothes. Wear what brings you the most warmth and joy.”*

**Appendix C**

Gift Card Membership Stimuli

*
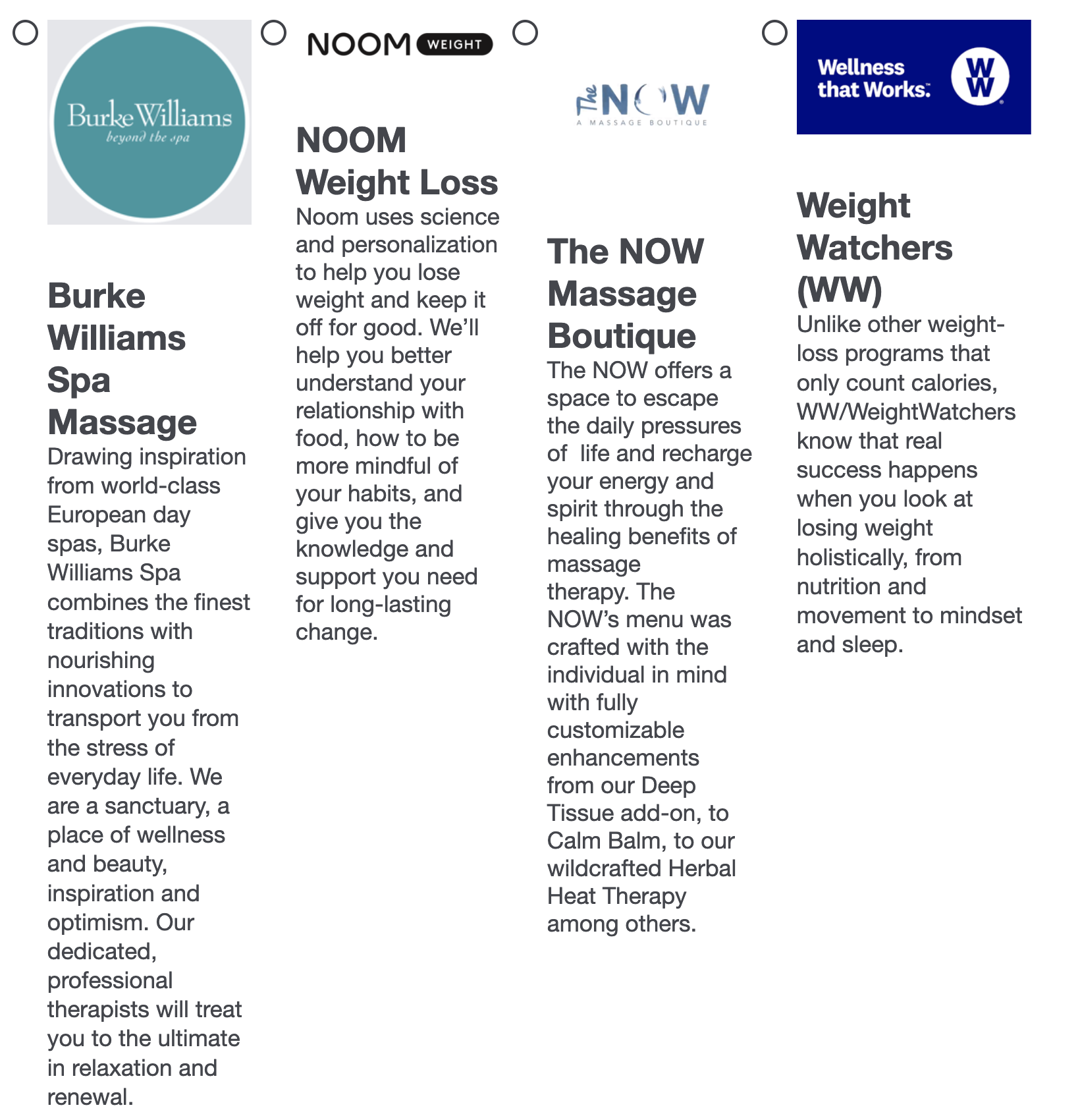
*

**Appendix D**

Mediation Analyses

Experiment 2 also explored the role of body shame as one potential mediator in a model linking LKM to feelings of empathy towards higher weight individuals. The proposed model draws upon previous research, which suggests that the more shame an individual feels about their own bodies, the greater their anti-fat attitudes are (Himmelstein & Tomiyama, 2015). Other work has shown that individuals with greater preoccupation with their physical appearances report greater anti-fat bias (O’Brien et al., 2007) and that internalized body shame may help explain the associations between anti-fat attitudes and denigrating “fat talk” (Webb et al., 2016). Taken together, an individual’s own negative self-perceptions may drive anti-fat bias such that reducing body shame may lead to greater empathy towards others and less weight stigma. We expected that lower body shame would mediate the relationship between the LKM condition and greater feelings of empathy towards higher weight individuals. We also hypothesized that greater empathy towards higher weight individuals would mediate the relationship between LKM and prosocial behavior towards higher weight individuals.

***LKM Intervention → Body Shame → Empathy***

Conditional on the assumption that body shame precedes empathy towards higher weight individuals, we ran a mediation analysis using 10,000 bootstrapped samples in Model 4 of PROCESS macro (Hayes A F, 2022) via SPSS software (v. 28) to obtain relative indirect effects of the LKM intervention on feelings of empathy through an individual’s own body shame. The control group was coded as “0” and treated as the reference group. Relative to the control group, the LKM intervention showed no significant effect on feelings of body shame, *b* = -0.05, *SE* = 0.23, *t* = -0.22*, p =* .828, 95% CI [-0.50, 0.40], nor was there a significant effect of body shame on feelings of empathy toward higher weight individuals, *b* = 0.003, *SE* = 0.08, *t* = 0.04*, p =* .969, 95% CI [-0.16, 0.16]. There was no observed significant indirect effect such that compared to the control group, lower levels of body shame among participants in the LKM intervention did not significantly predict levels of empathy,
(*a*_1_*b* = -0.0002, *SE* = 0.02, 95% *CI* [-0.04, 0.05]. However, the relative direct effect was significant, *b* = 0.50, *SE* = 0.24, *t* =2.10, *p* = .037, 95% CI [0.03, 0.97], suggesting that relative to the control group, the LKM intervention did affect feelings of empathy, controlling for an individual’s body shame. See Figure 1 for the mediation model. In other words, the variance in empathy was not accounted for by reported body shame as hypothesized in the proposal causal model. Given the limitations of testing a single mediator, we offer suggestions on testing alternative mediators in the discussion.


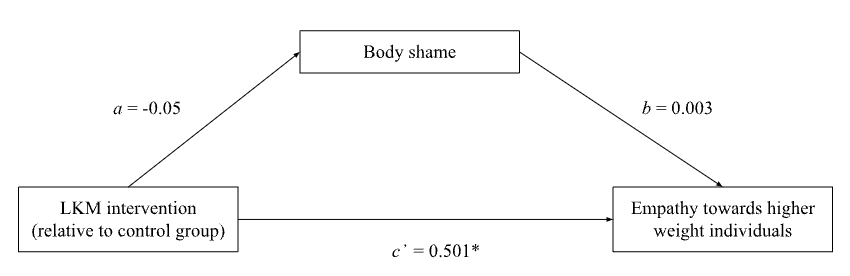


**Fig 1.** Mediation analysis for the effect of LKM on empathy via body shame. Significance of paths is denoted by **p* < .05. Body shame was not manipulated such that the proposed causal direction is assumed.

***LKM Intervention → Empathy → Prosocial Behavior***

Conditional on the assumption that feelings of empathy precede prosocial behavior, we ran a mediation analysis using 10,000 bootstrapped samples in Model 4 of PROCESS macro via SPSS software (v. 28) to obtain relative indirect effects on the dichotomous dependent variable of prosocial behavior (i.e., wellbeing vs. weight-loss gift card choice) for the indicator variables representing each condition (LKM, empathy, control). To better facilitate interpretation of the results, prosocial behavior was coded as “0” and treated as the reference group in this mediation analysis such that the outcome indicates greater stigmatizing behavior. Relative to the control group, the LKM intervention had a significant effect on feelings of empathy toward higher weight individuals, *b* = 0.53, *SE* = 0.24, *t* = 2.21*, p =* .028, 95% CI [0.06, 0.99], yet empathy, independent of condition, was not significantly related to prosocial behaviors toward higher weight individuals, *b* = -0.30, *SE* = 0.16, *Z*  = -1.80*, p =* .071, 95% CI [-0.62, 0.03]. The mediation analyses revealed no significant indirect effect such that compared to the control group, higher levels of empathy among participants in the LKM intervention did not significantly predict prosocial behavior, (*a*_1_*b* = -0.16, 95% CI [-0.47, 0.01]. In other words, the variance in prosocial behavior was not accounted for by feelings of empathy towards higher weight individuals as hypothesized in the proposed causal model. However, the relative direct effect was significant, *b* = 1.70, *SE* = 0.62, *Z* = 2.77, *p* = .006, 95% CI [0.50, 2.91], suggesting that relative to the control group, the LKM intervention was more likely to reward a weight loss gift card (i.e., engaging in weight stigmatizing behavior). See Figure 2 for the mediation model. We discuss the limitations of this single mediator model and offer further suggestions on alternative mediation models in the discussion.

***
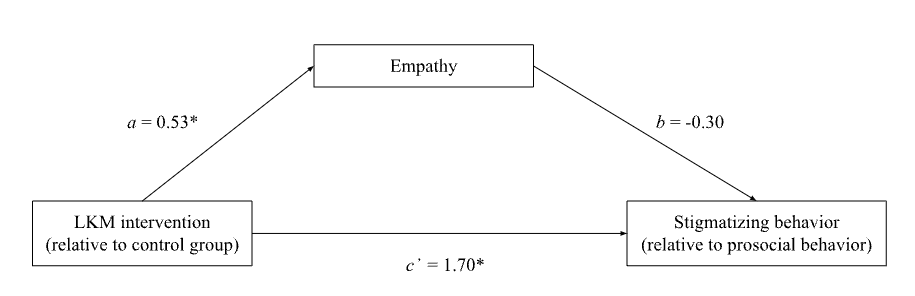
***

**Fig 2.** Mediation analysis for the effect of LKM on stigmatizing behavior via empathy. Significance of paths is denoted by **p* < .05. Empathy was not manipulated such that the proposed causal direction is assumed.

**Discussion on Mediation Models**

Contrary to our hypotheses, participants in the LKM condition did not report significantly lower body shame than those in the empathy intervention or control, nor did body shame predict feelings of empathy. There are several potential reasons for this. First, our measure of body shame may lack the temporal ordering to be considered a logical mediator to our criterion outcome of empathy, as body shame reflects a trait-like self-consciousness of one’s physical body, rather than an immediate antecedent to feelings of empathy. Body shame may therefore be more appropriately regarded as a predictor, rather than a mediator. Our own post-hoc analyses failed to find a significant association between body shame and anti-fat attitudes, which contradicts previous literature (Himmelstein & Tomiyama, 2015). Accordingly, it may be more fruitful to examine other psychosocial factors such as an individual’s fear of fat as a predictor of anti-fat prejudice (O’Brien et al., 2013). There are also more probable alternative mediators linking LKM to feelings of empathy. For example, guilt is an other-oriented emotion that may be more likely to engender an empathic compared to shame, which is a self-oriented emotional response that may hinder one’s ability to care for others response (Tangney et al., 2007).

Similarly, we did not find support for our proposed causal model linking the LKM intervention to prosocial behavior via feelings of empathy toward higher weight individuals, despite finding a significant direct effect of LKM on engaging in *more* stigmatizing behavior compared to the control group. While it is possible that the LKM intervention used in our study may lead to changes in untested, alternative mediators, it is important to note that our experimental manipulation of LKM and measurement of prosocial behavior using the gift cards were novel in their application towards higher weight individuals. Prior to testing alternative mediation models, it would be sensible to test the robustness of the brief LKM intervention and our measure of prosocial behavior in future replication studies.

1. We pre-registered a “Friend” vs “Stranger” condition for Experiment 1. As a manipulation check, we asked participants to indicate who they brought to mind in the “Friend” condition if they did not have a higher weight friend. We included all responses indicating a higher weight friend, family, or close acquaintance in the final analyses, and excluded any responses that indicated a lower weight individual. [↑](#footnote-ref-1)
